# Combining renal cell arrest and damage biomarkers to predict progressive AKI in patient with sepsis

**DOI:** 10.1186/s12882-021-02611-8

**Published:** 2021-12-15

**Authors:** Xiaolei Tao, Chunbo Chen, Weihong Luo, Jing Zhou, Jianwei Tian, Xiaobing Yang, Fan Fan Hou

**Affiliations:** 1 Division of Nephrology, Nanfang Hospital, Southern Medical University, National Clinical Research Center for Kidney Disease, State Key Laboratory of Organ Failure Research, Guangdong Provincial Clinical Research Center for Kidney Disease, Guangzhou Regenerative Medicine and Health-Guangdong Laboratory, 1838 North Guangzhou Ave, Guangzhou, 510515 China; 2 Department of Intensive Care Unit of Cardiovascular Surgery, Guangdong Cardiovascular Institute, Guangdong Provincial People’s Hospital, Guangdong Academy of Medical Sciences, Laboratory of South China Structural Heart Disease, Department of Critical Care Medicine, Guangdong Provincial People’s Hospital, Academy of Medical Sciences, The Second School of Clinical Medicine, Southern Medical University, Guangzhou, 510000 Guangdong China

**Keywords:** Sepsis, AKI, Progression, Risk prediction, Biomarker

## Abstract

**Background:**

Sepsis is the most common trigger for AKI and up to 40% of mild or moderate septic AKI would progress to more severe AKI, which is associated with significantly increased risk for death and later CKD/ESRD. Early identifying high risk patients for AKI progression is a major challenge in patients with septic AKI.

**Methods:**

This is a prospective, multicenter cohort study which enrolled adult patients with sepsis and initially developed stage 1 or 2 AKI in the intensive care unit from January 2014 to March 2018. AKI was diagnosed and staged according to 2012 KDIGO-AKI guidelines. Renal cell arrest biomarkers (urinary TIMP2*IGFBP7, u[TIMP-2]*[IGFBP7]) and renal damage biomarkers (urinary KIM-1[uKIM-1] and urinary IL-18 [uIL-18]) were measured at time of AKI clinical diagnosis, and the performance of biomarkers for predicting septic AKI progression alone or in combination were evaluated. The primary outcome was AKI progression defined as worsening of AKI stage. The secondary outcome was AKI progression with subsequent death during hospitalization.

**Results:**

Among 433 screened patients, 149 patients with sepsis and stage 1 or 2 AKI were included, in which 63 patients developed progressive AKI and 49 patients subsequently died during hospitalization. u[TIMP-2]*[IGFBP7], uKIM-1 and uIL-18 independently predicted the progression of septic AKI in which u[TIMP-2]*[IGFBP7] showed the greatest AUC (0.745; 95%CI, 0.667-0.823) as compared to uKIM-1 (AUC 0.719; 95%CI 0.638-0.800) and uIL-18 (AUC 0.619; 95%CI 0.525-0.731). Combination of u[TIMP-2]*[IGFBP7] with uKIM-1 improved the performance of predicting septic AKI progression with AUC of 0.752. u[TIMP-2]*[IGFBP7], alone or combined with uKIM-1/uIL-18, improved the risk reclassification over the clinical risk factor model alone both for the primary and secondary outcomes, as evidenced by significant category-free net reclassification index.

**Conclusions:**

Combination of renal cell arrest and damage biomarkers enhanced the prediction of AKI progression in patients with sepsis and improved risk reclassification over the clinical risk factors.

**Supplementary Information:**

The online version contains supplementary material available at 10.1186/s12882-021-02611-8.

## Background

Acute kidney injury (AKI) is a common complication in patients admitted to the intensive care unit (ICU), especially in those with sepsis [1]. Sepsis associated AKI accounts for approximately half of all AKI in ICU, which is associated with significantly increased risk for in-hospital death. Moreover, septic AKI is also associated with increased risk of later chronic kidney disease and end stage kidney disease [2].

AKI occurred in about 45-53% of patients with sepsis, and most septic AKI was mild or moderate AKI (KDIGO stage 1 or stage 2) [3, 4]. However, previous study showed that up to 40% these mild or moderate AKI would progress to more severe AKI (KDIGO stage 3), of which 30% required dialysis and the risk of death increased by 3-fold, as high as 70% [5]. Therefore, early identifying patients at high risk for progressive AKI might help clinicians to enhance individualized monitoring and personalized management in patient with septic AKI, which might prevent or halt the ongoing renal injury and improve the outcome of patients with sepsis.

Recently, there has been rising interest in searching and validating new biomarkers for early predicting AKI development and prognosis in different clinical settings. Renal cell cycle arrest biomarkers, urinary tissue inhibitor of metalloproteinases-2 (TIMP-2) and insulin-like growth factor binding protein-7 (IGFBP-7), have been shown to efficiently predict the risk of severe AKI development in ICU and were approved by U.S. FDA as a test of determining the risk of AKI development [6, 7]. It has been reported that renal tubular cells may produce and release TIMP-2 and IGFBP7 when exposed to cellular stress or injury, and may help renal cells maintain energy balance, prevent further DNA damage and division [7, 8]. But sustained renal cell cycle arrest will result in a senescent cell phenotype and lead to progressive injury [9]. A recent study reported that urinary [TIMP-2]*[IGFBP7] concentration at the early phase of septic shock was an independent factor to identify the population at high risk of progression from mild and moderate to severe AKI over the next 24 h with an AUC of 0.83 [5]. In addition, there were other novel renal injury biomarkers, such as kidney injury molecular-1 (KIM-1) and interleukin-18 (IL-18), which reflecting renal tubular damage and inflammation of AKI, also shown to predict the progression of AKI in the setting of ICU and cardiac surgery, and presented modest performance [10, 11]. To further improve the ability of biomarkers for predicting AKI progression in sepsis, carefully selecting and combining biomarkers might be a better approach for greater use. Compared with other AKI etiologies, septic AKI was thought to be associated with multi-mechanisms, such as renal microcirculation disorder, renal cell cycle stress, tubular injury and inflammation [1, 7, 12]. Combining renal cell arrest biomarkers with renal injury/inflammation biomarkers to predict the progression of septic AKI was not addressed before, and whether combining renal cell arrest and damage biomarkers could improve risk classification for progressive AKI in sepsis warrants further investigation.

We conducted a prospective, multicenter cohort study which included 149 adult septic patients who initially developed stage 1 or stage 2 AKI during ICU stay. Levels of novel urinary biomarkers ([TIMP-2]*[IGFBP7], KIM-1, and IL-18) were measured at time of AKI clinical diagnosis, and the utility of biomarkers for predicting septic AKI progression in combination was evaluated. Furthermore, the risk classification improvement of combining these biomarkers for predicting progressive septic AKI was investigated.

## Methods

### Study design and study population

We prospectively screened adult (age ≥ 18 years) patients who were admitted to the ICU in two academic teaching hospitals in China from January 2014 to March 2018. Eligible participants were patients who were admitted with sepsis and initially developed stage 1 or 2 AKI on admission or during hospitalization. The value of serum creatinine over a 6-month period before admission was used as baseline. Exclusion criteria included preexisting advanced CKD (baseline eGFR< 30 ml/min per 1.73m^2^) and a life expectancy less than 24 h. Patients with stage 3 AKI could not progress further and were excluded from analysis (Fig. S1).

AKI was diagnosed according to the Kidney Disease Improving Global Outcomes (KDIGO) Clinical Practice Guidelines for AKI based on serum creatinine criteria [13]. Not all patients in this study had precise records of urine output per hour, and we only used serum creatinine for AKI diagnosis and stages. Serum creatinine was measured once to twice per day to precisely define AKI and determine AKI progression. Sepsis was defined according to The Third International Consensus Definitions for Sepsis and Septic Shock [14]. This study was approved by the Institutional Review Board of the National Clinical Research Center for Kidney Disease and the Research Ethic Committee of Guangdong Provincial People’s Hospital, Guangdong Academy of Medical Sciences. This study was carried out in accordance with the code of ethics of the World Medical Association Declaration of Helsinki, and patients or the next of kin of the patients were informed and gave written informed consent.

### Procedures

All septic patients were treated according to Surviving Sepsis Campaign guidelines for management of severe sepsis and septic shock. Spot urine samples were collected daily for the first 14 days during hospitalization. Urine samples at the day of AKI clinical diagnosis were used for biomarker measuring. Urine samples were centrifuged at 3000 rpm for 10 min and the supernatants were stored at − 80 °C. Serum creatinine was measured on admission and twice a day during the first 5 days and at least daily thereafter. Clinical data for the study were collected from the hospital records, such as demographic, medication on admission, baseline renal function, Acute Physiology and Chronic Health Evaluation II (APACHE II) scores, Sequential Organ Failure Assessment (SOFA) scores, Multiple Organ Dysfunction Syndrome (MODS) scores, hemoglobin, blood urea nitrogen, serum albumin, blood lactate and procalcitonin. There was not any use of special membranes or cartridges in septic patients who received acute dialysis.

### Laboratory measurements

All biomarkers were measured in our central laboratory by standard protocols in a technician-blinded manner. The levels of renal cell arrest biomarkers, urinary TIMP-2*IGFBP7 (u[TIMP-2]*[IGFBP7]), were measured by ELISA kits (TIMP-2: DTM200, R&D Systems; IGFBP7: DY1334-05, R&D Systems) according to the manufacturer’s instructions. The levels of renal cell injury and inflammation biomarkers, urinary KIM-1 (uKIM-1) and urinary IL-18 (uIL-18), were measured by ELISA kits (KIM-1: DY1750B, R&D Systems; IL-18: ELH-IL18, RayBiotech) on the manufacturer’s instructions. All biomarkers were measured in triplicate and the intra- and inter- assay variability ranged 2–6% and 5–9%. Urinary albumin was quantified by an automatic analyzer and expressed as the ratio to urinary creatinine (UACR). All urinary biomarkers were normalized to urinary creatinine. Baseline eGFR was estimated by the CKD-Epidemiology Collaboration Eq. [15]. Levels of biomarkers measured on the day of initial AKI clinical diagnosis were used for all analysis.

### Outcome definitions

As previously reported [16, 17], the primary outcome was the progression of AKI, defined as worsening of KDIGO stage (from stage 1 to either stage 2 or stage 3, or from stage 2 to stage 3). Patients treated with acute dialysis at any point during hospitalization were defined as stage 3. The secondary outcome was AKI progression with death. Patients who died without AKI progression were excluded from the primary analysis because death may have been a competing risk for progression for these patients as previously reported [16, 17].

### Statistical analyses

We used the two-sample t test or the Mann-Whitney U test to compare continuous variables; and used the chi-squared/ Fisher exact test and categorical variables, respectively. All tests were two-tailed and *P* < 0.05 was considered significant. To evaluate the performance of u[TIMP-2]*[IGFBP7] for predicting septic AKI progression, in single or in combination with renal damage biomarkers or clinical risk factors, we used the conventional area under the receiver-operating characteristic (ROC) curve (AUC). We employed Logistic regression models to calculate the AUCs of urinary biomarkers in all analysis. To evaluate the utility of renal arrest and damage biomarkers on risk classification, we determined the category-free net reclassification improvement (NRI) and the integrated discrimination improvement (IDI), as previously described [18, 19].

## Results

### Cohort characteristics

A total of 433 patients admitted with sepsis in two hospitals were screened, and finally 149 patients with sepsis and stage 1 or 2 AKI were included for analysis (Fig. S1). Among 149 septic patients with AKI, 79 (53.0%) developed AKI on admission and 70 (47.0%) during hospitalization.

Among 149 patients with stage 1 or 2 AKI, 63 patients (42.3%) progressed to a higher stage of AKI during their hospitalization (32 individuals progressed to stage 2 and 31 progressed to stage 3); 23 of 63 (36.5%) progressors received acute dialysis; 45 of 63 (71.4%) developed AKI progression and subsequently died during hospitalization; 86 patients (57.7%) persisted in stage 1 or 2 AKI.

The characteristics 149 septic patients with or without AKI progression were showed in Table 1. Compared to those with AKI that did not progress, patients with AKI progression had lower proportion of male, more usage of nephrotoxic antibiotics before AKI diagnosis. AKI progressors had higher score of illness severity, such as the APACHE II, SOFA, and MODS scores (Table 1). There was no statistical difference in age, baseline renal function, serum albumin, levels of blood lactate and procalcitonin, and proportion of morbidities (hypertension, diabetes, and pre-CKD) on admission between patients with or without AKI progression.

**Table 1:** Characteristics of septic patients with and without AKI progression

Characteristics	Overall (*n* = 149)	Progression (*n* = 63)	Non-Progression (*n* = 86)	*P*
**Demographics**				
Age, y	59.6 ± 16.0	60.8 ± 15.2	58.8 ± 16.6	0.536
Male, n (%)	99 (66.4)	34 (54.0)	65 (75.6)	0.006
Hypertension, n (%)	67 (45.0)	28 (44.4)	39 (45.3)	0.913
Diabetes, n (%)	34 (22.8)	15 (23.8)	19 (22.1)	0.805
Prior CKD^a^, n (%)	15 (10.1)	8 (12.7)	7 (8.1)	0.361
**Mode of admission**				
Medical, n (%)	27 (18.1)	14 (22.2)	13 (15.1)	0.266
Surgical, n (%)	117 (78.5)	45 (71.4)	72 (83.7)	0.071
Emergency, n (%)	5 (3.4)	4 (6.3)	1 (1.2)	0.082
**Primary source of sepsis**				
Pulmonary, n (%)	90 (60.4)	48 (76.2)	42 (48.8)	0.001
Intra-abdominal, n (%)	27 (18.1)	9 (14.3)	18 (20.9)	0.345
Soft tissue, n (%)	6 (4.0)	2 (3.2)	4 (4.7)	0.651
Urinary, n (%)	4 (2.7)	2 (3.2)	2 (2.3)	0.751
Other, n (%)	40 (26.8)	14 (22.2)	26 (30.2)	0.276
**Baseline renal function**				
Serum creatinine, mg/dL	0.9 ± 0.3	0.9 ± 0.3	0.9 ± 0.3	0.861
eGFR, ml/min per 1.73m^2^	90.7 ± 27.0	87.8 ± 28.5	91.7 ± 25.9	0.429
**Parameters on ICU admission**				
APACHE II	20.0 (13.0-25.0)	23.0 (18.0-27.0)	17.0 (11.8-23.0)	< 0.001
SOFA	6.0 (4.0-8.0)	7.0 (5.0-9.0)	5.0 (4.0-7.0)	0.031
MODS	5.0 (3.0-6.0)	5.0 (4.0-7.0)	4.0 (3.0-6.0)	0.033
Hemoglobin, g/L	118.1 ± 78.1	106.7 ± 30.4	126.5 ± 98.8	0.014
Blood urea nitrogen, mmol/L	9.6 ± 7.4	10.6 ± 8.0	8.9 ± 6.9	0.274
Serum albumin, g/L	29.3 ± 7.7	28.2 ± 6.9	30.1 ± 8.1	0.072
Blood lactate, mmol/L	2.9 ± 2.1	3.3 ± 2.2	2.7 ± 2.0	0.086
Procalcitonin, ng/ml	2.2 (0.2-19.8)	2.1 (0.9-9.0)	2.6 (0.1-41.3)	0.900
**Nephrotoxic antibiotics**^b^**, n (%)**	49 (32.9)	28 (44.4)	21 (24.4)	0.010

Continuous variables were expressed as mean ± SD or median (25th percentile-75th percentile, interquartile range). Categorical variables were expressed as a number (%)

AKI progression is defined as worsening of AKI stage

^a^ Defined as baseline eGFR < 60 ml/min per 1.73m^2^. Baseline eGFR was calculated by CKD-Epidemiology Collaboration equation according to at least three measurements of serum creatinine over a 6-month period before admission

^b^ Usage of vancomycin, aminoglycosides, or amphotericin before AKI diagnosis

*Abbreviation*: *AKI* acute kidney injury, *CKD* chronic kidney disease, *ICU* intensive care unit, *eGFR* estimated glomerular filtration rate, *APACHE II* Acute Physiology and Chronic Health Evaluation II, *SOFA* Sequential Organ Failure Assessment, *MODS* Multiple Organ Dysfunction Syndrome

Table 2 compared the characteristics at time of AKI diagnosis and the in-hospital outcomes between patients with or without AKI progression. Patients with AKI progression had higher serum creatinine levels on the day of AKI diagnosis and greater increase of serum creatinine levels from the baseline at time of AKI diagnosis. Levels of renal cell arrest biomarker (u[TIMP-2]*[IGFBP7]) and damage biomarkers (uKIM-1 and IL-18) were significantly higher in patients with AKI progression as compared to those without. Patients with AKI progression had more adverse outcomes, such as receiving acute dialysis and in-hospital death, as compared with those without AKI progression (Table 2). In patients with AKI progression, the average timing from AKI initial diagnosis to serum creatinine peak was 2 days.

**Table 2:** Characteristics at time of AKI diagnosis in septic patients with and without AKI progression

Characteristics	Overall (n = 149)	Progression (n = 63)	Non-Progression (n = 86)	*P*
**AKI Severity**				
SCr at AKI diagnosis, mg/dL	1.6 ± 0.5	1.7 ± 0.5	1.5 ± 0.5	0.022
Peak SCr, mg/dL	1.8 ± 0.8	2.2 ± 0.9	1.6 ± 0.5	< 0.001
Change in SCr ^a^, mg/dL	1.0 ± 0.6	1.3 ± 0.7	0.7 ± 0.4	< 0.001
SCys-C at AKI diagnosis, mg/L	1.5 ± 0.7	1.7 ± 0.7	1.3 ± 0.7	0.004
AKI stage 1, n (%)	123 (82.6)	53 (84.1)	70 (81.4)	0.664
AKI stage 2, n (%)	26 (17.4)	10 (15.9)	16 (18.6)	0.664
AKI duration, d	2.0 (1.0-4.0)	3.5 (2.0-5.0)	1.0 (1.0-3.0)	< 0.001
**Biomarkers at time of AKI diagnosis**				
u[TIMP-2]*[IGFBP7], (μg/g Cr)^2^	1169.7 (426.6-3079.8)	2168.5 (1068.8-5274.9)	583.2 (293.6-1666.4)	< 0.001
uKIM-1, μg/g Cr	3.1 (1.5-6.0)	5.0 (2.7-7.3)	2.1 (0.8-4.8)	< 0.001
uIL-18, ng/g Cr	196.5 (79.5-664.5)	384.4 (89.4-1228.4)	131.5 (70.9-433.7)	0.017
uACR, mg/g Cr	138.3 (47.8-476.5)	221.5 (76.6-546.2)	108.8 (33.9-302.3)	0.006
**In-hospital outcomes**				
ICU stay, d	7.0 (4.0-12.0)	7.0 (4.5-12.0)	6.0 (3.0-12.0)	0.296
Acute dialysis, n (%)	23 (15.4)	23 (36.5)	0 (0.0)	< 0.001
In-hospital death, n (%)	45 (30.2)	45 (71.4)	0 (0.0)	< 0.001

AKI progression is defined as worsening of AKI stage

^a^ Serum creatinine level on the day of AKI diagnosis minus baseline serum creatinine level

*Abbreviation*: *SCr* serum creatinine, *SCys-C* serum cystatin C. u[TIMP-2]*[IGFBP7], urinary tissue inhibitor of metalloproteinase-2 and insulin-like growth factor-binding protein 7; uKIM-1, urinary kidney injury moleculer-1; uIL-18, urinary Interleukin-18; uACR, urinary albumin to creatinine ratio

### Performance of combining u[TIMP-2]*[IGFBP7] and renal damage biomarkers for predicting progressive AKI in Sepsis

Compared to those without AKI progression, patients with progressive AKI had significantly increased levels of u[TIMP-2]*[IGFBP7], uKIM-1, and uIL-18 at time of AKI clinical diagnosis (Table 2). As shown in Supplemental Table S1, u[TIMP-2]*[IGFBP7], uKIM-1 and uIL-18 predicted the progression of AKI in sepsis, with u[TIMP-2]*[IGFBP7] presented the greatest AUC (0.745, 95%CI 0.667-0.823) as compared to uKIM-1 (AUC 0.719, 95%CI 0.638-0.800) and uIL-18 (AUC 0.619, 95%CI 0.525-0.713). For predicting AKI progression with death, u[TIMP-2]*[IGFBP7] also showed the greatest AUC (0.777, 95%CI 0.700-0.854) as compared to uKIM-1 (AUC 0.738, 95%CI 0.653-0.822), and uIL-18 (AUC 0.657, 95%CI 0.557-0.758) (Supplemental Table S1).

Combining renal cell arrest biomarker (u[TIMP-2]*[IGFBP7]) with renal damage biomarkers (uKIM-1 and uIL-18) improved the performance for predicting AKI progression, with AUCs of 0.752 for u[TIMP-2]*[IGFBP7] with uKIM-1, and 0.747 for u[TIMP-2]*[IGFBP7] with uIL-18, respectively (Table 3). For predicting AKI progression with death, combining u[TIMP-2]*[IGFBP7] with uKIM-1 produced an increased AUC of 0.782, as compared to u[TIMP-2]*[IGFBP7] alone. However, combining u[TIMP-2]*[IGFBP7] with uIL-18 could not improve the performance for predicting AKI progression with death as compared to u[TIMP-2]*[IGFBP7] alone. Combining u[TIMP-2]*[IGFBP7] with UACR could not further improve the performance both for predicting AKI progression or AKI progression with death in sepsis (Table 3).

**Table 3:** Performance of renal arrest biomarkers for predicting septic AKI progression or AKI progression with death in single or combination with renal damage biomarkers

Outcomes	AUC	95% CI
**AKI progression**		
u[TIMP-2]*[IGFBP7]	0.745	0.667 to 0.823
u[TIMP-2]*[IGFBP7] + uKIM-1	0.752	0.675 to 0.828
u[TIMP-2]*[IGFBP7] + uIL-18	0.747	0.669 to 0.825
u[TIMP-2]*[IGFBP7] + uACR	0.745	0.668 to 0.823
u[TIMP-2]*[IGFBP7] + uKIM-1 + uIL-18 + uACR	0.755	0.679 to 0.832
**AKI progression with death**		
u[TIMP-2]*[IGFBP7]	0.777	0.700 to 0.854
u[TIMP-2]*[IGFBP7] + uKIM-1	0.782	0.705 to 0.859
u[TIMP-2]*[IGFBP7] + uIL-18	0.777	0.700 to 0.854
u[TIMP-2]*[IGFBP7] + uACR	0.778	0.700 to 0.855
u[TIMP-2]*[IGFBP7] + uKIM-1 + uIL-18 + uACR	0.780	0.703 to 0.857

AKI progression is defined as worsening of AKI stage

*Abbreviation*: *AUC* area under the receiver-operating characteristic curve, *CI* confidence interval; u[TIMP-2]*[IGFBP7], urinary tissue inhibitor of metalloproteinase-2 and insulin-like growth factor-binding protein 7; uKIM-1, urinary kidney injury moleculer-1; uIL-18, urinary Interleukin-18; uACR, urinary albumin to creatinine ratio

### Performance of combining u[TIMP-2]*[IGFBP7] with clinical risk factors for predicting progressive AKI in Sepsis

**Fig. 1:**
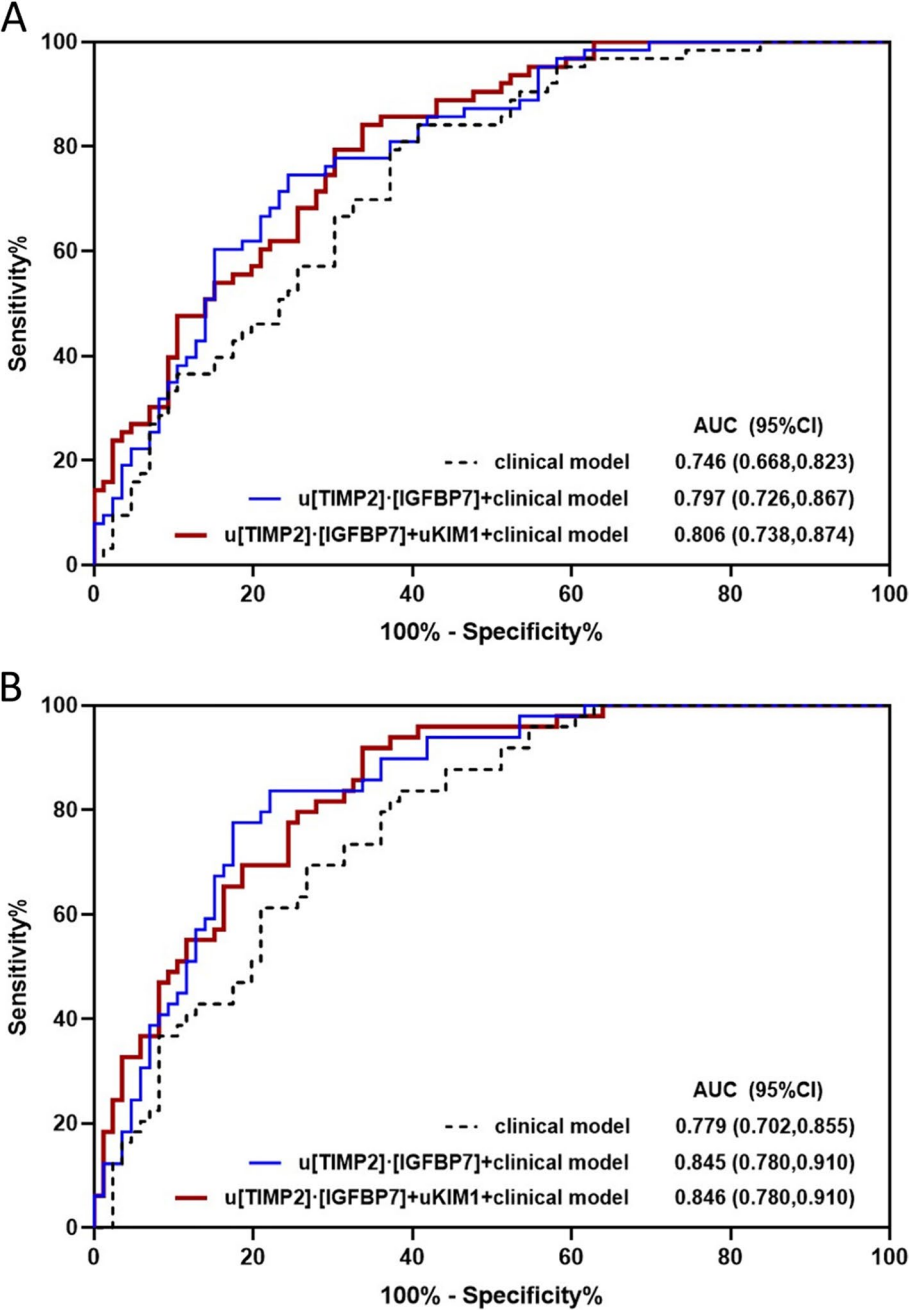
ROC analyses for predicting AKI progression or AKI progression with death. **A** The AUCs of renal cell arrest and damage biomarkers (uTIMP2*IGFBP7 and uKIM-1), and clinical model, at the time of AKI diagnosis, for predicting AKI progression. **B** The AUCs of renal cell arrest and damage biomarkers (uTIMP2*IGFBP7 and uKIM-1), and clinical model, at the time of AKI diagnosis, for predicting AKI progression with subsequent death

**Fig. 2:**
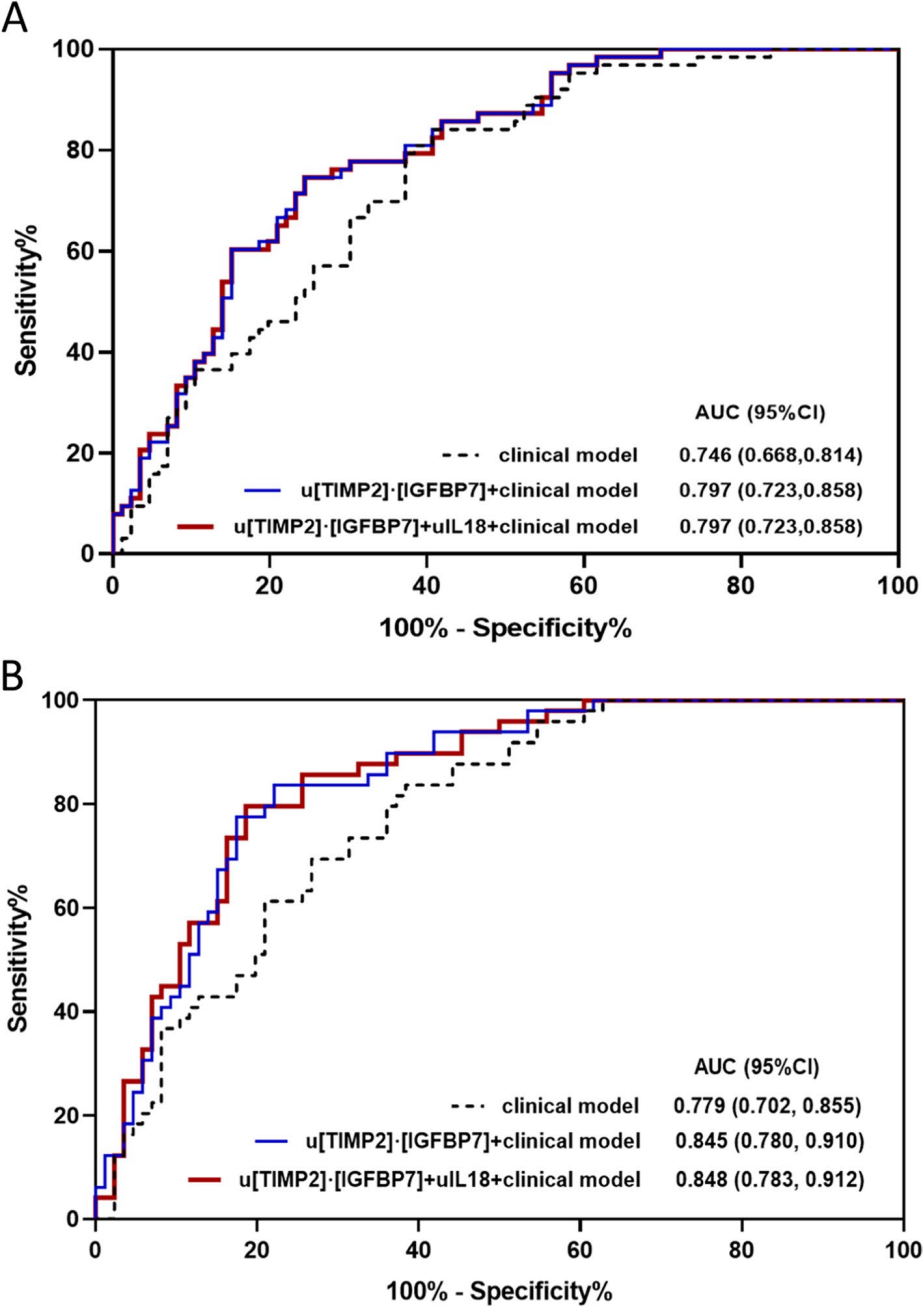
ROC analyses for predicting AKI progression or AKI progression with death. **A** The AUCs of renal cell arrest and inflammation biomarkers (uTIMP2*IGFBP7 and uIL18), and clinical model, at the time of AKI diagnosis, for predicting AKI progression. **B** The AUCs of renal cell arrest and inflammation biomarkers (uTIMP2*IGFBP7 and uIL18), and clinical model, at the time of AKI diagnosis, for predicting AKI progression with subsequent death

Combining u[TIMP-2]*[IGFBP7] with clinical risk factors, such as APACHE II and SOFA score, serum creatinine and Cys-C at time of AKI diagnosis, improved the performance for predicting septic AKI progression and AKI progression with death (Table 4). The clinical risk factor model comprised of age, gender, APACHE II, serum creatinine and albuminuria at time of diagnosis predicted the primary and secondary outcomes with AUCs of 0.746 (95%CI, 0.668-0.823) and 0.779 (95%CI, 0.702-0.855), respectively (Figs. 1 and 2). Combining u[TIMP-2]*[IGFBP7] with the clinical risk factor model further improved the AUCs to 0.797 (95%CI, 0.726-0.867) and 0.845 (95%CI, 0.780-0.910) as compared to clinical model alone both for predicting AKI progression or AKI progression with death. When combining both u[TIMP-2]*[IGFBP7] and uKIM-1 with the clinical model, the predicting performance further improved, with AUCs of 0.806 (95%CI, 0.738-0.874) and 0.846 (95%CI, 0.780-0.910) for the primary and secondary outcomes (Table 4 and Fig. 1). However, combination of u[TIMP-2]*[IGFBP7] and uIL-18 only improved the performance for secondary outcome (Fig. 2).

**Table 4:** Performance of renal arrest biomarkers for predicting septic AKI progression in single or combination with clinical risk factors

Outcomes	AUC	95%CI
**AKI progression**		
u[TIMP-2]*[IGFBP7]	0.745	0.667 to 0.823
u[TIMP-2]*[IGFBP7] + APACHE II	0.779	0.706 to 0.852
u[TIMP-2]*[IGFBP7]] + SOFA	0.752	0.675 to 0.829
u[TIMP-2]*[IGFBP7] + SCr at time of AKI diagnosis	0.752	0.675 to 0.829
u[TIMP-2]*[IGFBP7] + SCys-C at time of AKI diagnosis	0.754	0.677 to 0.831
u[TIMP-2]*[IGFBP7] + M^a^	0.797	0.726 to 0.867
u[TIMP-2]*[IGFBP7] + uKIM-1 + M	0.806	0.738 to 0.874
**AKI progression with death**		
u[TIMP-2]*[IGFBP7]	0.777	0.700 to 0.854
u[TIMP-2]*[IGFBP7] + APACHE II	0.828	0.760 to 0.897
u[TIMP-2]*[IGFBP7]] + SOFA	0.797	0.723 to 0.871
u[TIMP-2]*[IGFBP7] + SCr at time of AKI diagnosis	0.784	0.708 to 0.860
u[TIMP-2]*[IGFBP7] + SCys-C at time of AKI diagnosis	0.785	0.708 to 0.861
u[TIMP-2]*[IGFBP7] + M	0.845	0.780 to 0.910
u[TIMP-2]*[IGFBP7] + uKIM-1 + M	0.846	0.780 to 0.910

AKI progression is defined as worsening of AKI stage

^a^ M, clinical risk factor model. The clinical risk factor model for predicting AKI progression are comprised of age, gender, APACHE II, SCr at time of diagnosis, uACR at time of AKI diagnosis (AUC 0.746, 95% CI 0.668 to 0.823); The clinical risk model for predicting AKI progression with death are comprised of age, gender, APACHE II, SCr at time of diagnosis, uACR at time of AKI diagnosis (AUC 0.779, 95% CI 0.702 to 0.855)

*Abbreviation*: *AUC* area under the receiver-operating characteristic curve, *CI* confidence interval; u[TIMP-2]*[IGFBP7], urinary tissue inhibitor of metalloproteinase-2 and insulin-like growth factor-binding protein 7; APACHE II, Acute Physiology and Chronic Health Evaluation; SOFA, Sequential Organ Failure Assessment; SCr, serum creatinine; SCys-C, serum cystatin C; uKIM-1, urinary kidney injury moleculer-1

### Risk classification improvement of combining u[TIMP-2]*[IGFBP7] with renal damage biomarker or clinical risk factors

As shown in Table S2, adding u[TIMP-2]*[IGFBP7] to the clinical risk factor model resulted in the greatest improvement in risk reclassification both for the primary and the secondary outcomes, with a category-free net reclassification index (NRI) of 0.63 and 0.59 for AKI progression and AKI progression with death. Adding u[TIMP-2]*[IGFBP7] and uKIM-1 to the clinical risk factor model further improved risk classification over the clinical model alone, both for AKI progression and AKI progression with category-free NRI of 0.61 and 0.67, respectively (Table S2).

## Discussion

In this prospective, multicenter cohort study of adult patients with sepsis, we firstly showed that combining renal cell arrest biomarker and renal injury biomarkers could enhance the ability of biomarkers for predicting the progression of septic AKI. u[TIMP-2]*[IGFBP7], measured at time of AKI diagnosis, predicted both AKI progression and AKI progression with death in the setting of sepsis. Compared to u[TIMP-2]*[IGFBP7] alone, combination of u[TIMP-2]*[IGFBP7] with uKIM-1 slightly improved the performance for predicting both above outcomes, with AUC increased from 0.745 to 0.752 for AKI progression and from 0.777 to 0.782 for AKI progression with death. Moreover, we first showed that adding u[TIMP-2]*[IGFBP7] to the clinical risk factor model, alone or combined with renal injury biomarkers, significantly improved the risk classification of AKI progression and AKI progression with death in sepsis, as evidenced by significant NRI and IDI.

Sepsis was the most common trigger for AKI, septic patients were at the highest risk for developing AKI with an incidence ranged 22 -51% according to current KDIGO 2012 criteria [1, 20, 21]. Patients who developed mild or moderate AKI and subsequently progressed to severe AKI had the highest risk for death [7]. In our cohort, near 80% of sepsis patients with progressive AKI died during hospitalization, consistent with previous reports. Therefore, using novel biomarkers to enhance the risk classification of AKI progression upon clinical risk factors might help clinicians initiate close patient monitoring and plan appropriate management, which in turn might reduce the risk of death of these patients based on above additional prognostic information. Previous studies have showed that renal arrest biomarkers, u[TIMP-2]*[IGFBP7], predicted the progression of AKI in the setting of ICU and septic shock [5, 22–24]. Other novel renal injury or inflammation biomarkers, such as KIM-1, IL18, were also shown to predict progressive septic AKI [24–26], respectively. In this prospective study in patients with sepsis, we further directly compared the predictive performance of u[TIMP-2]*[IGFBP7] with the other novel injury/inflammation biomarkers in single or combination. Our results showed that combining u[TIMP-2]*[IGFBP7] with uKIM-1 could further improve the prediction of septic AKI progression compared to single biomarker prediction, which was also true for predicting AKI progression with death, suggesting that carefully selecting and combining biomarkers might be a better approach for greater application. Biomarkers of different type provides relevant information that improve their application and predictive value.

Albuminuria and serum creatinine are traditional markers of kidney injury. However, these existing markers had less sensitivity and specificity and were not sufficient for determining the risk of AKI progression [26–29]. Therefore, adding novel biomarkers to the clinical risk factor model which including albuminuria and serum creatinine would be a new way to increase risk assessment and stratification for AKI progression. The NRI denoted an improvement in reclassification as any increase in model-based predicted probabilities after the addition of the biomarker for events (AKI progression) and a decrease in probabilities for nonevents, and a large effect sizes had an NRI greater than 0.6 [30]. The results of our study have showed that adding u[TIMP-2]*[IGFBP7] to the clinical risk factor model could significantly improve risk classification for AKI progression alone or in combination with uKIM-1, with NRIs of 0.63 and 0.61 respectively. And this was also true for risk classification for the secondary outcome, i.e. AKI progression with death, with NRIs of 0.59 and 0.67. u[TIMP-2]*[IGFBP7], measured at time of septic AKI diagnosis, could not only be used as a tool assessing the risk of AKI progression in sepsis, but also provided additional prognostic information in hospital, such as subsequent death after AKI. Interestingly, combining u[TIMP-2]*[IGFBP7] with uKIM-1 and uIL-18 together could not significantly improve prediction of septic AKI progression as compared to u[TIMP-2]*[IGFBP7] with uKIM-1 combination. Whether this finding is due to modest ability of uIL-18 in unclear; however, it suggests that efficiently selecting and combining biomarkers for a multi-biomarker approach prediction needs more investigation. Furthermore, larger studies are warranted to explore the role of biomarkers in clinical practice, in order to entail advances in the management of septic patients and improve their outcomes.

### Strengths and limitations

Our study has the following strength. First, this was a multicenter, prospective cohort study. AKI and sepsis were diagnosed based on standardized criteria (KDIGO 2012 and sepsis-3) that are currently used in the international renal and critical care community. Second, we simultaneously measured well reported renal cell arrest biomarker and renal damage biomarkers and assessed the predictive performance and risk classification alone or combination with clinical risk factors in the setting of sepsis, which directly compared the predictive ability of biomarkers alone or in combination. This study also had limitations. Urinary creatinine excretion is not at a steady state during AKI; 24 h urinary excretion of biomarkers would be more meaningful. The number of primary outcomes was relatively small, and all patients were Chinese adults. Though this study showed an improvement of combining renal cell arrest and damage biomarkers to predict progressive AKI in patient with sepsis, terms of cost effectiveness, ease of the tests, and time consuming needed to be evaluated in a larger size patient population.

## Conclusions

Combination of renal arrest and damage biomarkers enhanced the prediction of AKI progression in patient with sepsis and improved risk reclassification over the clinical risk factor model alone. As this study was conducted in a pure sepsis population of ICU patients, our findings might have useful clinical implications for sepsis adults at risk for AKI progression.

## Supplementary Information

**Additional file 1: Table S1**. Performance of renal cell arrest and damage biomarkers for predicting septic AKI progression or AKI progression with death. **Table S2**. Analysis of risk reclassification of biomarkers over the clinical model for predicting AKI progression or AKI progression with death. **Figure S1**. Flow chart of patient enrollment and exclusion.

## Data Availability

The data used to support the findings of this study are available from the corresponding author upon reasonable request.

## Abbreviations

AKI: Acute kidney injury
ICU: Intensive care unit
TIMP-2: Tissue inhibitor of metalloproteinases-2
IGFBP-7: Insulin-like growth factor binding protein-7
KIM-1: Kidney injury molecular-1
IL-18: Interleukin-18
KDIGO: Kidney Disease Improving Global Outcomes
APACHE II: Acute Physiology and Chronic Health Evaluation II
SOFA: Sequential Organ Failure Assessment
MODS: Multiple Organ Dysfunction Syndrome
ROC: Receiver-operating characteristic
AUC: Area under the curve
NRI: Net reclassification improvement
IDI: Integrated discrimination improvement
