# Supplementary material for: Combining renal cell arrest and damage biomarkers to predict progressive AKI in patient with sepsis
Source: BMC Nephrol. 2021 Dec 15;22:415. doi: 10.1186/s12882-021-02611-8 (PMC8672478; doi:10.1186/s12882-021-02611-8)
Supplement: Supplementary file 1 — Additional file 1: Table S1. Performance of renal cell arrest and damage biomarkers for predicting septic AKI progression or AKI progression with death. Table S2. Analysis of risk reclassification of biomarkers over the clinical model for predicting AKI progression or AKI progression with death. Figure S1. Flow chart of patient enrollment and exclusion. [file 12882_2021_2611_MOESM1_ESM.pdf]

Supplemental Material

Table S1. Performance of renal cell arrest and damage biomarkers for predicting septic AKI progression or AKI progression with death

Outcomes	AUC	95%CI	*P*[a]
**AKI progression**			
u[TIMP-2]*[IGFBP7]	0.745	0.667 to 0.823	< 0.001
uKIM-1	0.719	0.638 to 0.800	< 0.001
uIL-18	0.619	0.525 to 0.713	0.013
uACR	0.631	0.541 to 0.720	0.004
**AKI progression with death**			
u[TIMP-2]*[IGFBP7]	0.777	0.700 to 0.854	< 0.001
uKIM-1	0.738	0.653 to 0.822	< 0.001
uIL-18	0.657	0.557 to 0.758	0.002
uACR	0.642	0.547 to 0.737	0.004

[a] Compared with 0.5.

AUC, area under the receiver-operating characteristic curve; CI, confidence interval; u[TIMP-2]*[IGFBP7], urinary tissue inhibitor of metalloproteinase-2 and insulin-like growth factor-binding protein 7; uKIM-1, urinary kidney injury moleculer-1; uIL-18, urinary Interleukin-18; uACR, urinary albumin to creatinine ratio.

Table S2. Analysis of risk reclassification of biomarkers over the clinical model for predicting AKI progression or AKI progression with death

Outcomes	Category-Free NRI (95% CI)	*P*	NRI in Progressors (95% CI)	*P*	NRI in Non-progressors (95% CI)	*P*	IDI (95% CI)	*P*
**AKI progression**								
Clinical risk factors	referent		referent		referent		referent	
Clinical risk factors +u[TIMP-2]*[IGFBP7]	0.63 (0.31-0.94)	<0.001	0.30 (0.06-0.54)	0.015	0.33 (0.12-0.53)	0.002	0.08 (0.04-0.13)	<0.001
Clinical risk factors +uKIM-1	0.53 (0.21-0.86)	0.001	0.30 (0.06-0.54)	0.015	0.23 (0.02-0.44)	0.030	0.09 (0.04-0.14)	<0.001
Clinical risk factors +uIL-18	0.31 (-0.02-0.64)	0.059	0.14 (-0.11-0.39)	0.260	0.16 (-0.05-0.38)	0.132	0.02 (0.00-0.05)	0.048
Clinical risk factors +u[TIMP-2]*[IGFBP7]+uKIM-1	0.61 (0.29-0.93)	<0.001	0.33 (0.09-0.57)	0.007	0.28 (0.07-0.49)	0.009	0.11 (0.05-0.16)	<0.001
**AKI progression with death**								
Clinical risk factors	referent		referent		referent		referent	
Clinical risk factors +u[TIMP-2]*[IGFBP7]	0.59 (0.25-0.94)	<0.001	0.27 (-0.01-0.54)	0.063	0.33 (0.12-0.53)	0.002	0.12 (0.06-0.18)	<0.001
Clinical risk factors +uKIM-1	0.56 (0.21-0.90)	0.002	0.35 (0.08-0.62)	0.014	0.21 (0.00-0.42)	0.052	0.10 (0.05-0.16)	<0.001
Clinical risk factors +uIL-18	0.50 (0.15-0.85)	0.002	0.22 (-0.06-0.51)	0.117	0.28 (0.07-0.49)	0.009	0.05 (0.01-0.10)	0.006
Clinical risk factors +u[TIMP-2]*[IGFBP7]+uKIM-1	0.67 (0.33-1.01)	<0.001	0.35 (0.08-0.62)	0.014	0.33 (0.12-0.53)	0.002	0.13 (0.07-0.19)	<0.001

AKI progression is defined as worsening of AKI stage.

Clinical risk factors for AKI progression are comprised of age, gender, APACHE II, SCr at time of diagnosis, uACR at time of AKI diagnosis.

Abbreviation:

NRI, net reclassification improvement; IDI, integrated discrimination improvement. SCr, serum creatinine; uACR, urinary albumin to creatinine ratio.

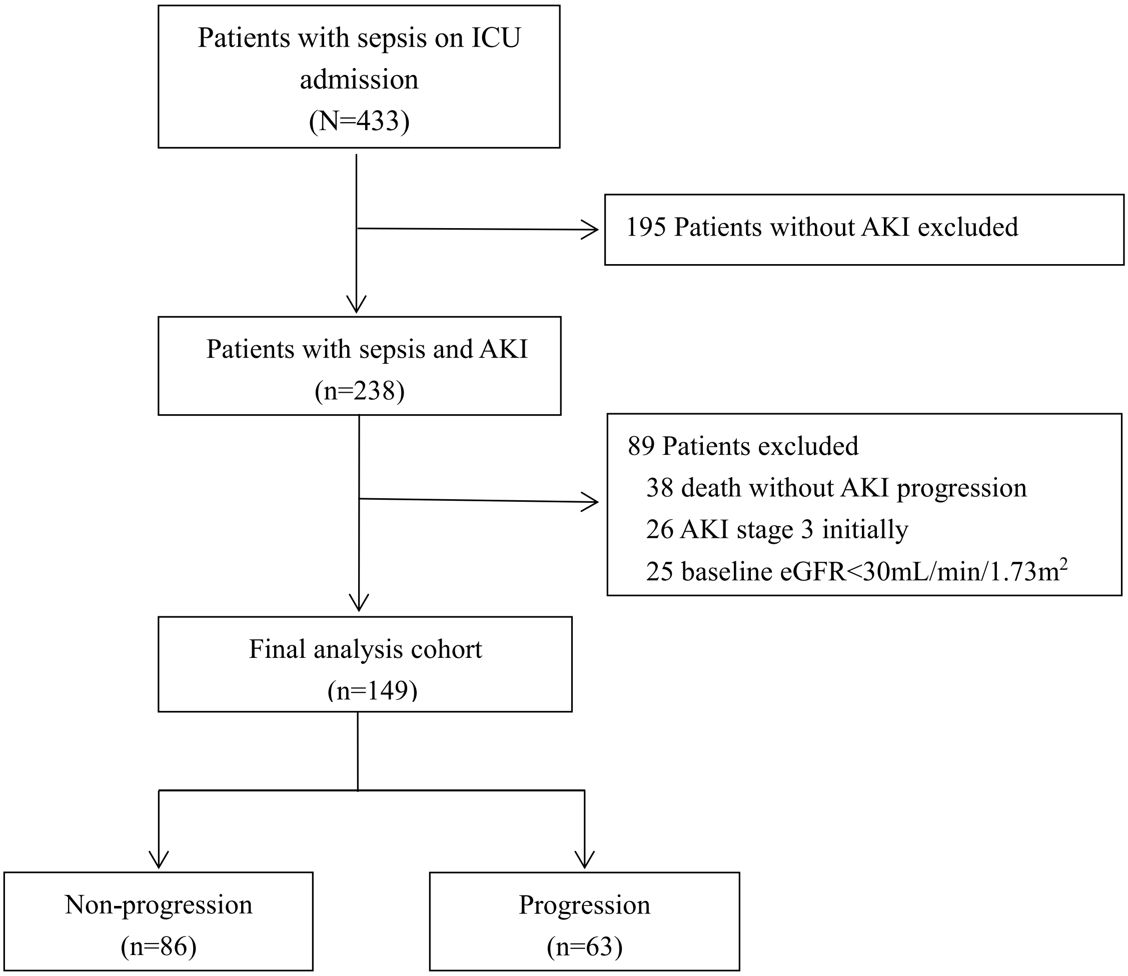

Figure S1. Flow chart of patient enrollment and exclusion.
